# Detection and Neutralization of SARS-CoV-2 Using Non-conventional Variable Lymphocyte Receptor Antibodies of the Evolutionarily Distant Sea Lamprey

**DOI:** 10.3389/fimmu.2021.659071

**Published:** 2021-06-21

**Authors:** Leslie Y. T. Leung, Srijit Khan, Patrick Budylowski, Zhijie Li, Sofiya Goroshko, Yanling Liu, Shilan Dong, James R. Carlyle, James M. Rini, Mario Ostrowski, Götz R. A. Ehrhardt

**Affiliations:** ^1^ Department of Immunology, University of Toronto, Toronto, ON, Canada; ^2^ Department of Medicine, University of Toronto, Toronto, ON, Canada; ^3^ Department of Biochemistry, University of Toronto, Toronto, ON, Canada; ^4^ Department of Molecular Genetics, University of Toronto, Toronto, ON, Canada

**Keywords:** SARS-CoV-2, monoclonal antibody, sea lamprey (*Petromyzon marinus*), variable lymphocyte receptor, neutralization

## Abstract

SARS-CoV-2 is a newly emerged betacoronavirus and the causative agent for the COVID-19 pandemic. Antibodies recognizing the viral spike protein are instrumental in natural and vaccine-induced immune responses to the pathogen and in clinical diagnostic and therapeutic applications. Unlike conventional immunoglobulins, the variable lymphocyte receptor antibodies of jawless vertebrates are structurally distinct, indicating that they may recognize different epitopes. Here we report the isolation of monoclonal variable lymphocyte receptor antibodies from immunized sea lamprey larvae that recognize the spike protein of SARS-CoV-2 but not of other coronaviruses. We further demonstrate that these monoclonal variable lymphocyte receptor antibodies can efficiently neutralize the virus and form the basis of a rapid, single step SARS-CoV-2 detection system. This study provides evidence for monoclonal variable lymphocyte receptor antibodies as unique biomedical research and potential clinical diagnostic reagents targeting SARS-CoV-2.

## Introduction

Coronavirus disease 19 (COVID-19) emerged from Wuhan, China, in December 2019 and progressed into a global pandemic in 2020, with over 160 million cases and over 3 million deaths documented worldwide by the World Health Organization (1). The disease is caused by the severe acute respiratory syndrome coronavirus 2 (SARS-CoV-2), a recently emerged betacoronavirus closely related to bat coronaviruses (2). Cell entry is mediated by the interaction of the receptor binding domain (RBD) of the viral spike (S) protein with the angiotensin-converting enzyme 2 (ACE2) receptor on the target cell, a process further facilitated by the proteolytic processing of the S-protein by the TMPRSS2 serine protease (3). Epitopes located on the viral S-protein RBD are targeted by most neutralizing antibodies isolated from COVID-19 convalescent or active patients (4–7). Consequently, the viral S-protein features prominently in multiple vaccination strategies and in antibody-based therapeutic approaches (8). The ready accessibility of the S-protein on the viral surface indicates that it may also represent a leading target for antibody-based virus diagnostics. While conventional antibodies recognize a wide variety of antigens with high degree of specificity, they are typically only poorly reactive with certain antigens such as carbohydrate posttranslational protein modifications, a feature that contributes to glycan shielding as immune evasion mechanism of viral pathogens, including those of the family *coronaviridae* (9–11). Exploring non-conventional antibody platforms for SARS-CoV-2 S-protein reactive antibodies is therefore a promising strategy to isolate reagents aimed at exploiting vulnerabilities of the virus that conventional antibodies may not readily recognize.

The variable lymphocyte receptor (VLR) molecules of the jawless sea lamprey *(Petromyzon marinus)* represent an alternative antibody system with a repertoire estimated to exceed 10^14^ clonotypes (12–14). Three VLR genes (*VLRA*, *B*, and *C*) encode antigen receptors that are expressed on the surface of cells resembling mammalian αβ T cells (15), B cells (16), and γδ T cells (17), respectively, and, in the case of VLRB, molecules are secreted as multimeric complexes (18). Unlike the immunoglobulin (Ig)-fold of conventional antibodies, the use of the leucine-rich repeat (LRR) as the basic structural unit of VLR antibodies results in a solenoid shape with a flexible, highly variable loop protruding from the capping C-terminal LRR. A gene conversion-like process leads to the assembly of a mature VLR gene consisting of a N-terminal capping LRR (LRR-NT), followed by an LRR1 module, 0-9 highly diverse LRRv units, a LRRve unit, a connecting peptide (CP), the capping C-terminal LRR (LRR-CT), and an invariant stalk (19). Structural analyses of VLR antibodies in complex with their respective antigens, exemplified by VLR RBC36 (20) and VLRB.2D (21), revealed that VLR antibody-antigen interactions are mediated by residues lining the concave surface of the VLR antibody and by residues located in the C-terminal loop. These antigen recognition characteristics are distinct from those of conventional antibodies and indicate that VLR antibodies may recognize epitopes that conventional immunoglobulins may not readily bind, a notion supported by various monoclonal VLR antibodies with unique protein and carbohydrate antigen binding characteristics (18, 22–25). Furthermore, VLR antibodies display a high degree of thermostability and retain antigen binding capacity under a wide range of pH and ionic strength conditions (18), indicating that they may represent a robust alternative complementing conventional antibodies for the detection of viral pathogens. Here we report the isolation and characterization of monoclonal VLRB antibodies that specifically recognize the S-protein of SARS-CoV-2 but not those of related coronaviruses. We further provide evidence for efficient neutralization of SARS-CoV-2 by monoclonal VLRB antibodies and demonstrate rapid, single-step detection of SARS-CoV-2 using VLRB antibodies, highlighting the application potential of these unique reagents for diagnostic and therapeutic targeting of SARS-CoV-2.

## Materials and Methods

### Cells and Reagents

Human embryonic kidney (HEK) 293T and 293F cells were grown in Dulbecco’s modified Eagle Medium supplemented with 5% fetal bovine serum (FBS), glutamine, and penicillin/streptomycin (100U/ml). Cells were maintained at 37°C with 5% CO_2_ in a humidified atmosphere. Sea lamprey larvae were obtained from the United States Geological Survey (Great Lakes Science Center, Millersburg, MI). Fluorescently labeled antibodies and streptavidin were obtained from SouthernBiotech (Birmingham, AL) and Novus Biologicals (Littleton, CO) and HRP labeled streptavidin was obtained from Cell Signaling Technology (Danvers, MA). Ni-NTA resin for protein purification was obtained from Qiagen (Hilden, Germany) and TMB ELISA substrate from ThermoFisher Scientific (Waltham, MA). Polyclonal rabbit anti-HCoV-OC43 antibodies were purchased from MyBioSource (San Diego, CA) and monoclonal anti-SARS-CoV-2 clone RBD-2D1 was obtained from mAb Lab Biotech (Toronto, ON). Anti-HCoV-229E (clone 9.8E12) (26) and anti-SARS-CoV (clone CR3022) (27) antibodies were purified in our laboratories. Soluble trimeric S-proteins of SARS-CoV-2, SARS-CoV, and HCoV-OC43 were cloned into the doxycycline inducible expression vector PB-T-PAF and expressed in HEK293F cells, followed by purification of recombinant protein from culture supernatants using Ni-affinity chromatography and size exclusion chromatography as described earlier (28, 29). The quality of the purified trimeric S-proteins was monitored by negative stain electron microscopy. SARS-CoV-2 S-protein variants B.1.117 and B.1.351 were obtained from InvivoGen (San Diego, CA) and re-cloned into the PB-T-PAF expression vector (28) prior to use in transient transfection assays. Variable gene sequences for anti-SARS-CoV2 monoclonal antibody 4A8 (30) were custom synthesized and recombinant antibodies were generated as described previously (31). Human serum from a SARS-CoV-2 vaccinated individual (ChAdOx1, sample collected 4 week after vaccination) was obtained with informed consent according to the Declaration of Helsinki and Research Ethics Board approval.

### Lamprey Larvae Immunization and Isolation of Monoclonal VLRB Antibodies

All animal experiments were performed with approval of the Animal Care Committee (Research Oversight and Compliance Office, Division of the Vice-President, Research and Innovation, University of Toronto). Six lamprey larvae (10cm in length) were anesthetized by immersion in MS-222 (Sigma-Aldrich, St. Louis, MO) and were immunized by intracoelomic injection of 1x10^7^ paraformaldehyde-fixed Jurkat T cells coated with the recombinant trimeric ectodomain of SARS-CoV-2 S-protein chemically crosslinked to the carrier cells using 1-ethyl-3-(3-dimethylaminopropyl)carbodiimide hydrochloride (ThermoFisher Scientific (Waltham, MA) without addition of adjuvants. Animals received a booster immunization after 2 weeks and circulating lamprey lymphocytes were harvested after an additional 10 days. Total RNA was extracted using RNeasy spin columns (Qiagen, Hilden, Germany) and VLRB transcripts were amplified by RT-PCR without sequences encoding the signal peptide and C-terminal stalk regions, followed by gap-repair cloning into the pCTC-ON2 vector using *S cerevisiae* strain EBY100 (32). Yeast cells harboring SARS-CoV-2 S-protein reactive clones were enriched by two rounds of magnetic bead purification using biotinylated SARS-CoV-2 S-protein and anti-biotin antibodies followed by a final round of purification using FACS sorting of reactive yeast cells using the biotinylated ectodomain of SARS-CoV-2 S-protein and detection using phycoerythrin (PE) labeled streptavidin. Alternatively, we generated SARS-CoV-2 tetramers by coupling biotinylated SARS-CoV-2 S-protein to streptavidin-PE at a calculated molar ratio of 4:1 for one hour on ice followed by addition of excess biotin to block potentially unoccupied biotin binding sites. Enrichment of SARS-CoV-2 S-protein-reactive EBY100 clones was performed using anti-PE magnetic beads (**Supplementary Figure 1**).

### Generation of Recombinant SARS-CoV-2 Reactive Monoclonal VLRB Antibodies

VLRB sequences were retrieved from EBY100 transformants and cloned into the p367HH vector modified to contain signal peptide sequences in addition to the HA- and 6xHis epitope tags and the C-terminal VLRB stalk region (33). VLRB expression plasmids were transiently transfected into HEK293T cells using polyethyleneimine (PEI) (34) at a ratio of 3µg PEI:1µg DNA and VLRB containing culture supernatants were harvested 48 hours later. Subsequently, HEK293F ‘Spike’ cells expressing the full length SARS-CoV-2 S-protein containing two mutations (K986P, V987P) stabilizing the protein in a prefusion conformation and three amino acid exchanges targeting the S1/S2 protease cleavage site (R682S, R683S, R685S) were used to validate reactivity of monoclonal VLRB antibodies as previously described (35). Briefly, cells were incubated with VLRB-containing culture supernatants for 20 minutes on ice, followed by incubation with monoclonal murine anti-VLRB antibodies (clone 4C4) (12) and PE-labeled anti-mouse IgG secondary antibodies. VLRB reactivity was assessed by flow cytometry using a Guava easyCyte HT instrument (EMD Millipore). Sequences of monoclonal VLRB antibodies consistently reactive with S-protein-expressing cells were determined by DNA sequencing to avoid potential further analyses of identical, independently isolated clones. Candidate clones were selected for large-scale protein purification using Ni-affinity chromatography.

### VLRB Antibody Specificity Analysis

HEK293F cells were plated in 24-well or 12-well plates at a concentration of 275,000 cells/ml (275,000 cells/well for 24-well plates, 550,000 cells/well for 12-well plates) overnight prior to transfection. The following day, expression plasmids encoding the S-proteins of SARS-CoV-2, SARS-CoV, HCoV-OC43 (OC43), or HCoV-229E (229E), or SARS-CoV-2 variants B.1.1.7 or B.1.351, were individually co-transfected with plasmids encoding GFP at a 4:1 S-protein:GFP plasmid ratio using the PEI method described above, with 3µg PEI:1µg DNA. Cells were removed from the plates 48h post-transfection using PBS/0.5mM EDTA. Subsequently, cells were incubated with purified monoclonal VLRB antibodies for 25 minutes on ice, followed by incubation with monoclonal murine anti-VLRB antibodies (clone 4C4) and PE-labeled goat-anti-mouse IgG secondary antibodies. To verify cell surface expression of the S-proteins, cells were incubated with the respective S-protein reactive antibodies, followed by incubation with PE-labeled secondary antibodies. VLRB reactivity and S-protein expression were assessed by flow cytometry using a Guava easyCyte HT instrument (EMD Millipore). Dead cell exclusion was performed by addition of propidium iodide (1µg/ml) and transfected cells were identified by expression of GFP (gating strategy and cell surface expression are depicted in **Supplementary Figure 2**). Detection of SARS-CoV-2 wild-type and variant S-proteins with human serum was performed at a 1:1000 serum dilution followed by detection of bound antibodies with PE-conjugated anti-human Ig secondary reagents. For VLRB antibody specificity evaluation by ELISA, 96-well plates were coated with recombinant trimeric S-proteins of SARS-CoV-2, SARS-CoV, or HCoV-OC43, recombinant RBD of SARS-CoV-2, or bovine serum albumin (BSA) at 4°C for 2 days. Plates were then blocked with 5% milk in PBS for 2h at room temperature. Purified monoclonal VLRB antibodies were 10-fold serially diluted in 1% milk in PBS and incubated on the plates at 4°C overnight. Plates were washed 3 times with PBS prior to incubation with monoclonal anti-VLRB antibodies (clone 4C4) for 2h at room temperature. Plates were washed for another 3 times with PBS, followed by incubation with HRP-conjugated goat-anti-mouse secondary antibodies for 1h at room temperature. Plates were washed 5 times with PBS and developed with TMB substrates for 15 minutes at room temperature. Reactions were terminated with 2M sulfuric acid. Plates were read at OD 450nm using a SpectraMax i3 instrument (Molecular Devices, San Jose, CA) and analyzed following subtraction of background readings obtained in wells incubated with PBS/1% milk.

### SARS-CoV-2 S-Protein RBD and Antibody Competition Assays

For the SARS-CoV-2 S-protein RBD competition assays, VLRB B7, B33, or B39, or the murine antibody clone RBD-2D1 was pre-incubated with recombinant RBD at 1:1 and 1:10 antibody:RBD ratios in PBS/0.5%BSA on ice for 10-15 minutes prior to incubation HEK293F Spike cells. Subsequently, cells were incubated with anti-VLRB antibodies (clone 4C4) and goat-anti-mouse secondary antibodies. For the antibody competition assays, VLRB B7, B33, or B39 were pre-incubated with murine antibody clone RBD-2D1 at 1:1 and 1:10 VLRB : RBD-2D1 ratios in PBS/0.5%BSA for 10-15 minutes on ice. The mixtures were then incubated with HEK293F Spike cells for 25 minutes on ice, followed by incubation with monoclonal anti-His antibodies as the recombinant VLRB antibodies contain a 6xHis epitope tag on the invariant stalk. Dead cell exclusion was performed by the addition of propidium iodide (1µg/ml) and flow cytometric analyses of antibody signals were performed as described above.

### Inhibition of SARS-CoV-2 S-Protein and RBD Binding to ACE2-Expressing HEK293F Cells

VLRB antibodies were 5-fold serially diluted with PBS/0.5%BSA, then incubated with biotinylated recombinant S-proteins or RBD for 10-15 minutes on ice. Subsequently, the mixtures were added to HEK293F cells stably expressing the ACE2 receptor for 20 minutes on ice. After two washes with PBS/0.5%BSA, cells were incubated with streptavidin-PE for 20 minutes on ice. Dead cell exclusion was performed by addition of propidium iodide (1µg/ml) and S-protein binding was assessed by flow cytometry using a Guava easyCyte HT instrument (EMD Millipore).

### SARS-CoV-2 Neutralization

VeroE6 cells were plated in 96-well plates at a concentration of 30,000 cells/well 24 hours prior to viral transduction. The following day, SARS-CoV-2 strain SB2-P4-PB clone 1 (36) at 100xTCID_100_ was pre-incubated with two-fold dilution series of recombinant VLRB antibodies for one hour at 37°C followed by addition to VeroE6 cells in quadruplicate and inoculation for an additional hour. Subsequently, the viral inoculum was replaced with culture medium. Cultures were assessed for virus-induced cytopathic effects including rounded, refractile cell morphology and detachment from tissue culture plates after 5 days of culture and the number of CPE-positive or CPE-negative wells determined, thereby resulting in values of 0%, 25%, 50%, 75% or 100% neutralization for each VLRB antibody concentration. Curves were fitted using non-linear sigmoidal regression and IC50 values were calculated using the GraphPad Prism 6.0 software package. All neutralization studies using authentic SARS-CoV-2 were performed under Biosafety Level 3 conditions in accordance with the requirements of the Office of Environmental Health and Safety of the University of Toronto.

### SARS-CoV-2 Detection Using VLRB Tetramers

Monomeric VLRB antibodies were generated by cloning VLRB B39 and VLRB B7 antibody sequences lacking sequences encoding the 56 C-terminal amino acids into the p367HH-AVI vector backbone, which provides the HA and 6xHis epitope tags as well as the recognition sequence of the BirA biotin ligase (37). Recombinant proteins were generated in HEK293T cells, purified using Ni-affinity chromatography and *in vitro* biotinylation was performed as described previously (37). VLRB tetramers were generated by incubating biotinylated monomeric VLRB protein in PBS/1% BSA with streptavidin-HRP for 25 minutes on ice, followed by addition of free biotin at a concentration of 100µM to block potentially unoccupied biotin binding sites on streptavidin-HRP.

Membrane fragment enriched cell lysates of HEK293F Spike and HEK293F ACE2 cells were generated by incubating 2x10^7^ cells in 2ml of hypotonic buffer (10mM NaCl, 5mM MgCl_2_, 10mM Tris pH8.0) with protease inhibitors followed by cell disruption using 10 strokes of a Dounce homogenizer with B-type pestle and 3 sonication pulses. Cell lysates were centrifuged for 10 minutes at 500xg to remove nuclei and remaining intact cells and lysates were frozen in aliquots at -80°C. For detection of S-protein containing membrane fragments or SARS-CoV-2 viral particles, 65 µg of membrane-enriched cell lysates or serial dilutions of SARS-CoV-2 viral particles starting at 2x10^7^ TCID_50_/ml in 50µl were incubated with streptavidin-HRP VLRB tetramers for 10 minutes at room temperature, then added to anti-SARS-CoV-2 antibody (clone RBD-2D1) coated plates and incubated for 20 minutes. Subsequently, the plates were washed five times with PBS and bound tetramers were assayed by the addition of TMB substrate and quenching of the reaction by addition of an equal volume of 2M sulfuric acid. HRP enzymatic activity was determined at OD 450nm and analyzed following subtraction of background readings obtained in well incubated with PBS/1% BSA.

### Statistical Analyses

Statistical analyses were performed using Friedman and Kruskal-Wallis tests using the GraphPad Prism 6.0 software package (San Diego, CA) and the R Stats Package (version 4.0.2).

## Results

### VLRB Antibodies B7, B33, and B39 Recognize the S-Protein of SARS-CoV2

**Figure 1:**
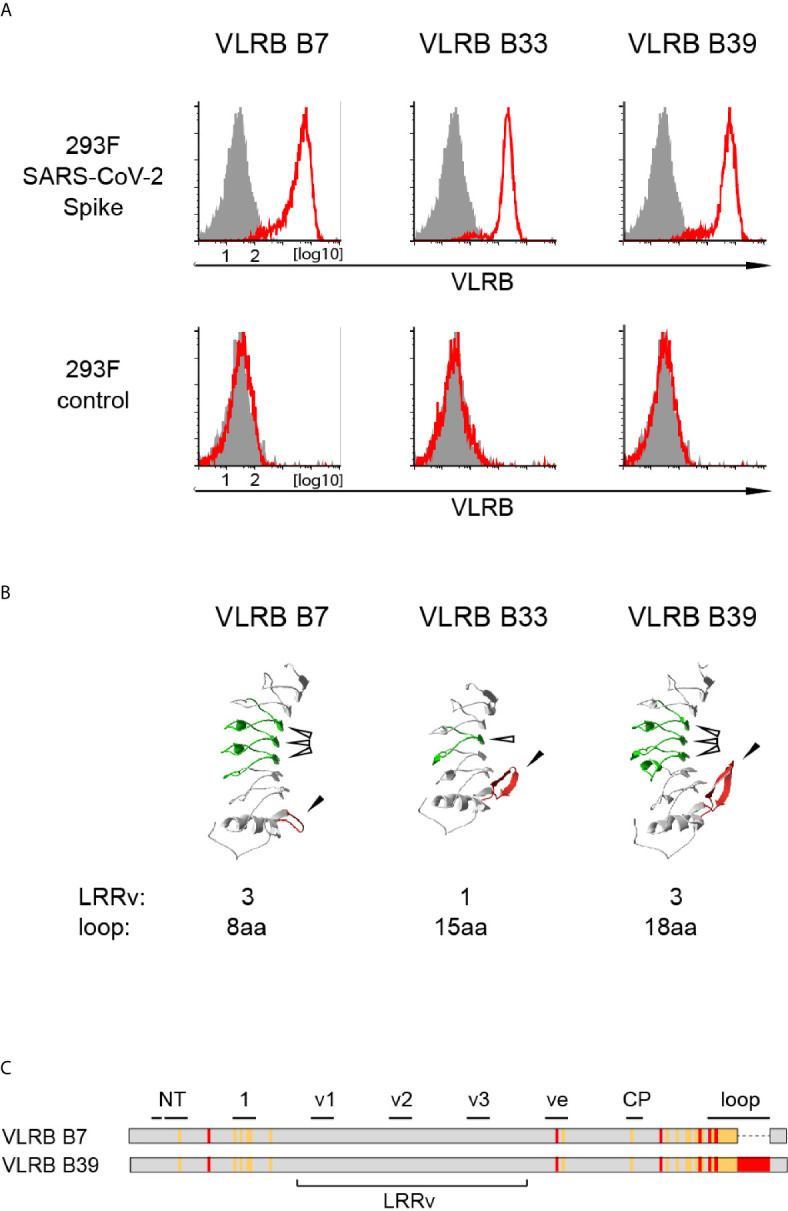
Isolation of SARS-CoV-2 S-protein reactive monoclonal VLRB antibodies. **(A)** Recognition of HEK293F cells stably expressing SARS-CoV-2 S-protein (top row) but not negative control untransfected HEK293F cells by selected monoclonal VLRB antibodies. VLRB antibody binding is shown by open red histograms and signals obtained with secondary antibody only as negative control are depicted by filled grey histograms. Shown is a representative of at least six independent experiments. **(B)** Structural characteristics of selected monoclonal VLRB antibodies. LRRv cassettes are shown in green (indicated by open arrowheads) and sequences encoding the C-terminal loop are depicted in red (indicated by closed arrowheads). Protein models were generated using the Phyre2 protein modeling platform (38). Numbers of LRRv units incorporated into the VLRB antibodies and loop lengths of the VLRB antibodies are indicated. **(C)** Graphic representation of amino acid sequences of VLRB B7 and VLRB B39. Identical residues are shown in grey, conserved differences by orange lines and non-conserved differences by red lines. Black bars indicate residues forming parallel β-sheets located in the predicted antigen binding sites of the capping N-terminal LRR (NT), LRR1 (1), LRRv (v1-v3), LRRve (ve), connecting peptide (CP), or the C-terminal loop protruding from the capping C-terminal LRR (loop).

Lamprey larvae respond readily to immunizations with particulate antigens, including primary cells or cell lines (22, 23, 33). In order to generate VLRB antibodies against the S-protein of SAR-CoV-2, we immunized lamprey larvae with Jurkat carrier cells coupled with recombinant trimeric SARS-CoV-2 S-protein. Antisera from immunized animals or non-immunized control animals were collected for verification of reactivity to the SARS-CoV-2 S-protein by ELISA. SARS-CoV-2 S-protein reactive polyclonal antisera were obtained from 5 out of 6 immunized animals (**Supplementary Figure 2**). Following enrichment for S-protein reactive cells using a yeast surface display approach, we isolated 3 monoclonal VLRB antibodies, VLRB B7, VLRB B33, and VLRB B39, which consistently recognized HEK293F Spike cells expressing SARS-CoV-2 S-protein containing two stabilizing proline mutations (K986P, V987P) and three amino acid exchanges targeting the S1/S2 protease cleavage site (R682S, R683S, R685S) but not untransfected HEK293F control cells (**Figure 1A**). Sequence analysis revealed that VLRB B7 contained 3 LRRv segments but only a short C-terminal loop whereas VLRB B33 and VLRB B39 contained longer C-terminal loop sequences and 1 and 3 LRRv units, respectively (**Figure 1B**). Interestingly, the LRRv units of VLRB B7 and VLRB B39 were identical whereas the remaining capping LRR-NT and LRR-CT as well as the LRR1, LRRve, and CP sequences showed numerous differences, particularly in residues lining the predicted antigen binding regions (**Figure 1C**). These findings demonstrate the potential of the non-conventional VLR antibody platform to generate antibodies reactive to the S-protein of SARS-CoV-2.

### VLRB Antibodies B7, B33, and B39 Specifically Bind the S-Protein of SARS-CoV2, but Not the S-Proteins of Related Coronaviruses

**Figure 2:**
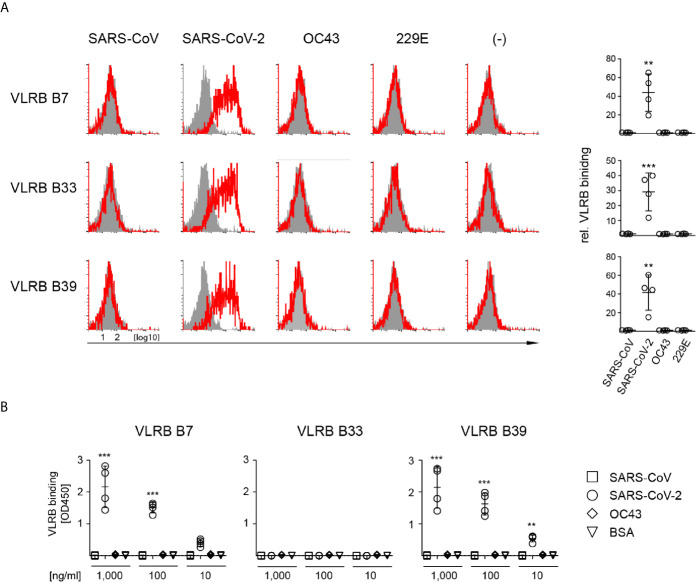
Specific recognition of SARS-CoV-2 S-protein by monoclonal VLRB antibodies. **(A)** HEK293F cells transiently co-transfected with expression plasmids encoding the indicated wt S-proteins and GFP were analyzed for S-protein binding by flow cytometry. Open histograms depict representative experiments for S-protein recognition by VLRB B7, VLRB B33, and VLRB B39. Closed histograms show signals with secondary antibodies only. Scatter plots depict MFI values normalized to negative control. Statistical significance was determined using Friedman tests (n = 5) and is indicated by asterisks (**) for p < 0.01, (***) for p < 0.001. **(B)** Recognition of recombinant trimeric S-proteins by VLRB antibodies by ELISA. Scatter plots depict OD450 values following background subtraction. Statistical significance was determined using Friedman tests (n = 5) and is indicated by asterisks (**) for p < 0.01, and (***) for p < 0.001.

Next, we sought to determine whether VLRB B7, B33, and B39 are reactive only to the S-protein of SARS-CoV-2 or whether they are cross-reactive with the S-proteins of other coronaviruses. Using a flow cytometric approach, we transiently transfected HEK293F cells with plasmids encoding the S-proteins of SARS-CoV, SARS-CoV-2, HCoV-OC43, or HCoV-229E and assayed reactivity of cells positive for the co-transfected EGFP marker. These experiments demonstrated that VLRB B7, VLRB B33, and VLRB B39 specifically recognized the SARS-CoV-2 S-protein (**Figure 2A**). Importantly, the experiments were performed with wild type (wt) SARS-CoV-2 S-protein expression constructs, unlike the initial screening steps and experiments, which were performed with the recombinant trimeric stabilized SARS-CoV-2 S-protein mutants. In an independent approach, we tested the specificity of the monoclonal VLRB antibodies by ELISA. Plates coated with the trimeric S-proteins of SARS-CoV-2, SARS-CoV, or OC43 were incubated with 10-fold serial dilutions of the VLRB B7, B33, and B39 antibodies. Similar to the preceding experiment using flow cytometry, VLRB B7 and VLRB B39 specifically recognized the SARS-CoV-2 S-protein (**Figure 2B**). Unexpectedly, VLRB B33 did not recognize the SARS-CoV-2 S-protein by ELISA, despite providing robust signals in flow cytometry assays. These experiments establish SARS-CoV-2 specificity of the VLRB B7, B33, and B39 antibodies.

### VLRB Antibodies B7, B33, and B39 Differentially Recognize S-Protein Variants of SARS-CoV-2

**Figure 3:**
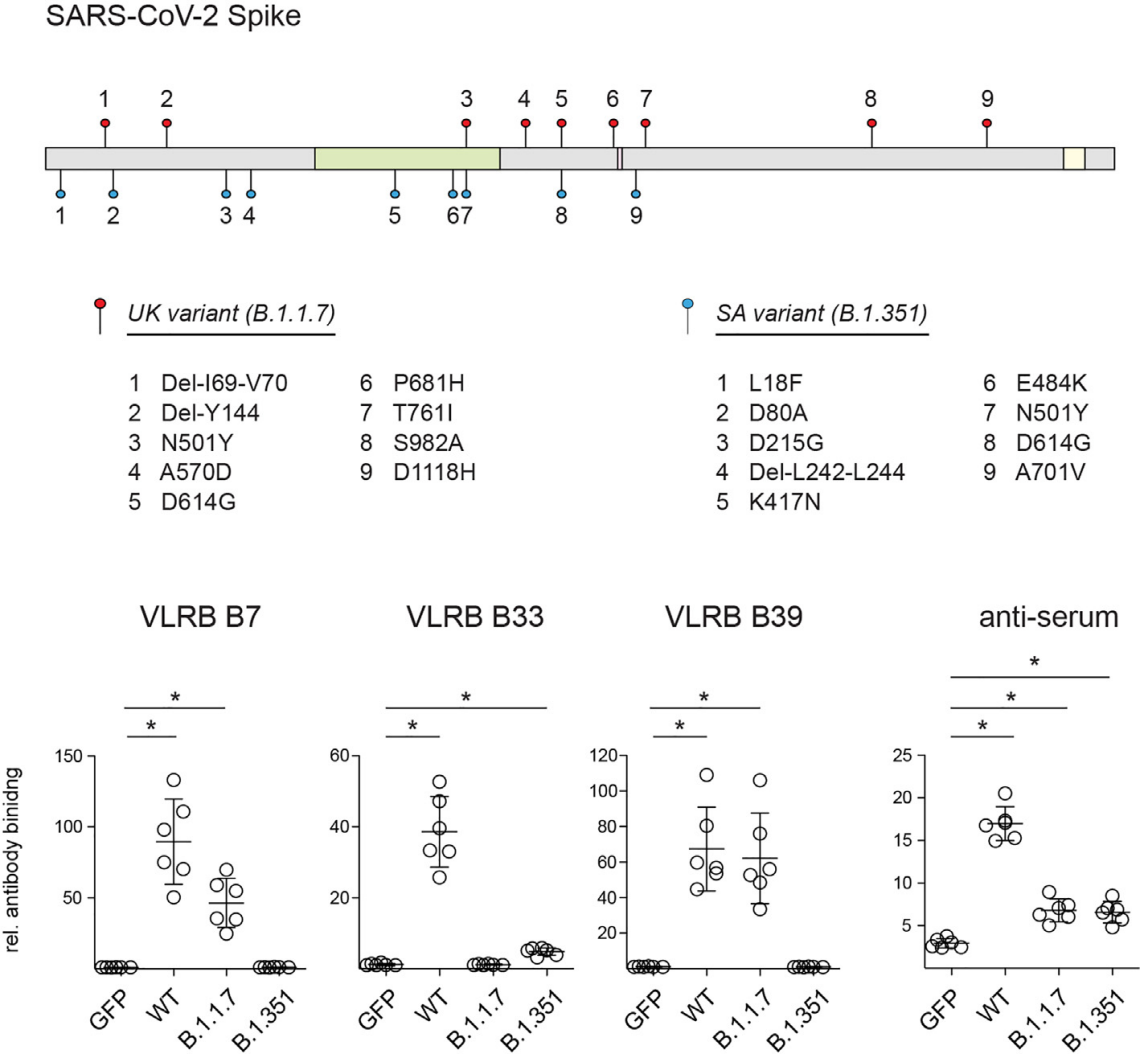
Recognition SARS-CoV-2 S-protein variants by monoclonal VLRB antibodies. Graphic illustration depicts mutations found in the SARS-CoV-2 B.1.1.7 (filled red circles) and B.1.351 (filled blue circles) variants. Regions encoding the RBD (green), proteolytic cleavage site (maroon) and transmembrane domain (yellow) are indicated. HEK293F cells transiently co-transfected with expression plasmids encoding the indicated wt and variant S-proteins and GFP were analyzed for S-protein recognition by VLRB antibodies or control human serum 4 weeks following ChAdOx1 vaccination by flow cytometry. Open circles represent MFI values normalized to negative control cells incubated only with secondary antibodies. Horizontal bars indicate mean +/- SD (n = 6). Statistical significance was determined using Kruskal-Wallis tests with Holms-Sidak *post hoc* analysis and is indicated by asterisks (*) for p < 0.05.

During the course of COVID-19 pandemic, various variants of concern of SARS-CoV-2 emerged with non-synomymous mutations in the viral genome including sequences encoding the S-protein that may alter viral transmissibility and recognition by the adaptive immune system (39–41). To explore potential differential recognition of S-protein variants, we transfected HEK293F cells with expression plasmids encoding the sequence of the wt S-protein, the B.1.1.7 variant first detected in the UK, or the B.1.351 variant first detected in South Africa, and we analyzed S-protein recognition by VLRB B7, B33, or B39. These experiments demonstrated that VLRB B7 and B39 recognized the B.1.1.7 variant but not the B.1.351 variant (**Figure 3**). Conversely, we observed consistent binding with reduced signal intensity of VLRB B33 to the B.1.351 variant compared to wild-type S-protein transfected cells whereas no binding was observed to cells expressing the B.1.1.7 variant. Control experiments in which transfected cells were analyzed using anti-serum from a SARS-CoV-2 vaccinated individual showed cell surface expression of SARS-CoV-2 wt and variant S-proteins. These experiments demonstrate that monoclonal VLRB antibodies can be used to discriminate between SARS-CoV-2 wt and variant antigens.

### VLRB B7 and VLRB B39 Recognize Epitopes Located Within the wt SARS-CoV-2 S-Protein RBD

**Figure 4:**
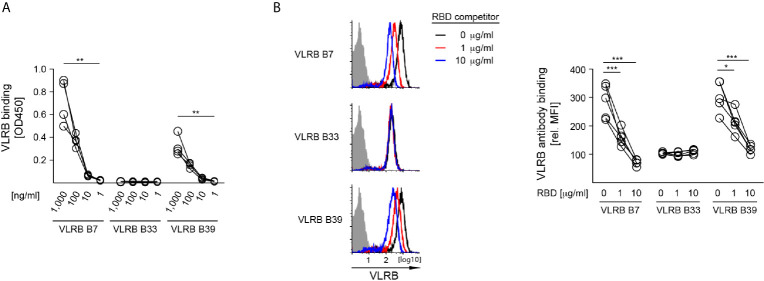
SAR-CoV-2 S-protein RBD recognition by VLRB B7 and VLRB B39, but not VLRB B33. **(A)** Recognition of recombinant SARS-CoV-2 S-protein RBD by VLRB antibodies by ELISA. Scatter plots depict OD450 values of four independent experiments following background subtraction at the indicated VLRB antibody concentrations. **(B)** VLRB binding to HEK293F Spike cells was assessed in the presence or absence of the indicated concentrations recombinant SARS-CoV-2 S-protein RBD. Open histograms depict representative experiments for S-protein recognition by VLRB B7, VLRB B33 and VLRB B39. Closed histograms show signals with secondary antibodies only. Scatter plots depict MFI values normalized to negative control. Statistical significance for (**A**, n = 4), (**B**, n = 5) was determined using Friedman tests and is indicated by asterisks (*) for p < 0.05, (**) for p < 0.01, and (***) for p < 0.001.

After confirming the specificity of VLR B7, B33, and B39 to the wt SARS-CoV-2 S-protein, we next sought to determine whether these VLR antibodies recognized the RBD or other epitopes elsewhere on the SARS-CoV-2 S-protein. In ELISA assays where plates were coated with recombinant wt SARS-CoV-2 S-protein RBD and incubated with 10-fold serially diluted VLRB antibodies, both VLR B7 and B39 recognized the RBD in a concentration dependent manner (**Figure 4A**). As expected from earlier experiments, VLRB B33 did not show any RBD reactivity. Independently, we explored whether recombinant RBD protein was able to block VLRB antibody binding to HEK293F Spike cells. Pre-incubation of the VLRB antibodies with various concentration of RBD protein followed by flow cytometric assessment of VLRB antibody binding revealed that VLRB B7 and VLRB B39 binding was inhibited by pre-incubation with RBD protein whereas VLRB B33 binding was unaffected (**Figure 4B**). Collectively, these results suggest that VLR B7 and B39 recognize the RBD of SARS-CoV-2 S-protein, whereas VLR B33 recognizes an epitope outside of the RBD.

### VLRB B7 and VLRB B39 Neutralize wt SARS-CoV-2

**Figure 5:**
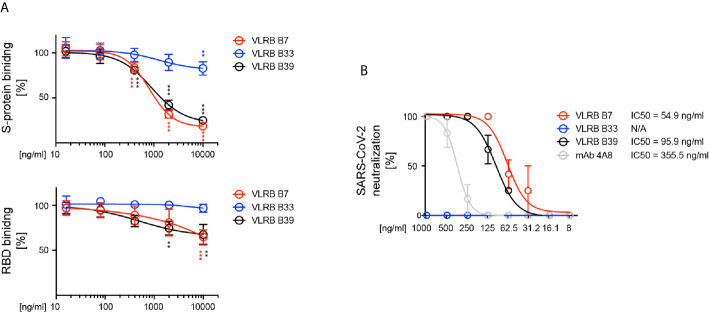
Neutralization of SARS-CoV-2 by VLRB B7 and VLRB B39. **(A)** Inhibition of SARS-CoV-2 (top panel) or SARS-CoV-2 RBD (bottom panel) binding to huACE2 by VLRB B7 and VLRB B39. Biotinylated trimeric S-protein was pre-incubated with the indicated amounts of monoclonal VLRB antibodies for 10 min prior to addition to HEK293F cells expressing huACE2 and assessment of S-protein binding by flow cytometry. Symbols indicate median (+/- SD) of MFI values normalized to negative control. Statistical significance was determined using Friedman tests (n = 5) and is indicated by asterisks (**) for p < 0.01, and (***) for p < 0.001. **(B)** Neutralization of SARS-CoV-2 by VLRB B7 and VLRB B39. 100xTCID_100_ of SARS-CoV-2 preparations were pre-incubated with the indicated amounts of VLRB antibodies prior to addition to Vero E6 cultures. IC50 values were determined after 5 days of culture. Recombinant monoclonal antibody 4A8 was used as positive control.

The observed RBD-recognition of VLRB B7 and VLRB B39 prompted us to investigate the SARS CoV-2 neutralization potential of these VLRB antibodies. In an initial series of surrogate neutralization experiments, we explored whether pre-incubation of biotinylated wt trimeric SARS-CoV-2 S-protein or biotintylated RBD with the VLRB antibodies would interfere with S-protein or RBD binding to HEK293F cells expressing the ACE2 receptor. Flow cytometric detection of bound S-protein demonstrated that VLR B7 and B39 could inhibit the binding S-protein to ACE2-expressing cells in a concentration dependent manner (**Figure 5A**). Weak inhibition of S-protein binding by pre-incubation was also observed for VLRB B33, albeit only at very high concentrations of 10µg/ml. Similarly, we observed inhibition of RBD binding to ACE2 expressing cells following pre-incubation with VLRB B7 or VLRB B39, but not VLRB B33 although the inhibitory activity was reduced compared to experiments using the complete trimeric S-protein (**Figure 5A**, bottom panel). Following observed inhibitory activity of VLRB B7 and VLRB B39 in the surrogate viral neutralization assay, we explored *in vitro* inhibition of SARS-CoV-2 infection of VeroE6 cells by these VLRB antibodies. Pre-incubation of 100 TCID with 2-fold serial dilutions of VLRB B7, VLRB B33, or VLRB B39 revealed potent neutralization activity of VLRB B7 and VLRB 39 with half-maximum inhibitory concentration (IC50) values of 54.9ng/ml and 95.9ng/ml, respectively (**Figure 5B**). This compares with an observed IC50 value of 355.5ng/ml for control antibody 4A8, a monoclonal antibody isolated from a convalescent COVID-19 patient with reported IC50 values of 610ng/ml and 390ng/ml determined by viral CPE and viral RNA assessment, respectively (30). Conversely, VLRB B33 did not demonstrate any *in vitro* neutralization activity. Together, these results highlight the neutralization potential of monoclonal VLRB antibodies.

### VLRB B39 Tetramers Allow for Rapid Detection of wt SARS-CoV-2

**Figure 6:**
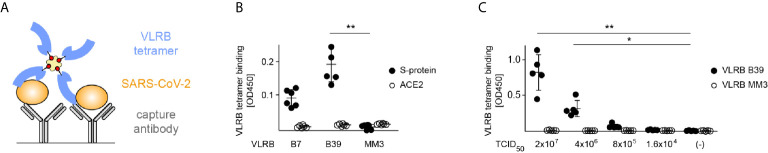
Single-step detection of SARS-CoV-3 by VLRB B39 tetramers. **(A)** Graphic representation of the VLRB antibody-based SARS-CoV-2 detection. **(B)** Detection of SARS-CoV-2 S-protein by VLRB tetramers. Signals obtained from cell lysates of HEK293F Spike cells and negative control HEK293F ACE2 cells are depicted in filled and open circles, respectively. Shown are absorption values at 450nm following background subtraction. Mean values (+/- SD) are indicted by horizontal bars. **(C)** Specific recognition of SARS-CoV-2 by VLRB B39 tetramers. Signals obtained for SARS-CoV-2 detection by VLRB B39 tetramers are shown in filled black circles and signals obtained with negative control VLRB MM3 tetramers in open circles. Mean values (+/- SD) are indicted by horizontal bars. Statistical significance was determined using Friedman tests (**B**, n = 6), (**C**, n = 5) and is indicated by asterisks (*) for p < 0.05, and (**) for p < 0.01.

The observed highly specific recognition of SARS-CoV-2 S-protein by the isolated VLRB antibodies suggested an application potential for virus detection. Earlier, we developed VLRB tetramers by site-specific biotinylation of recombinant VLRB protein using the BirA biotin ligase followed by coupling of the biotinylated VLRB antigen binding units to fluorescently labeled streptavidin (37). We modified this system by coupling biotinylated SARS-CoV-2 reactive VLRB antigen binding units to streptavidin-HRP. Incubation of these VLRB tetramers with virus samples, followed by addition to capture plates coated with the SARS-CoV-2-specific murine monoclonal RBD-2D1 antibody form the basis for a single-step assay that allows the direct detection of the captured antigen (**Figure 6A**). While the monoclonal antibody RBD-2D1 recognizes an epitope located within the viral S-protein RBD, the binding of RBD-2D1 does not interfere with the binding of VLRB B7, VLRB B33, or VLRB B39 to the SARS-CoV-2 S-protein (**Supplementary Figure 4**).

To simulate membrane embedded S-protein of viral particles we generated membrane fragment enriched cell lysates from HEK293F Spike cells and negative control HEK293F ACE2 cells as sources of antigen. Streptavidin-HRP tetramers containing biotinylated antigen binding domains of VLRB B39 but not of the negative control VLRB MM3, a monoclonal VLRB antibody recognizing human CD38 ectoenzyme in an activation dependent manner (22), allowed the rapid and specific detection of SARS-CoV-2 S-protein (**Figure 6B**). In contrast, these experiments did not result in any detectable signals using lysates from HEK293F ACE2 cells. VLRB B7 tetramers also resulted in readily detectable signals in combination with HEK293F Spike cells lysates; however, the signal intensity was noticeably weaker compared to VLRB B39 tetramers and did not reach statistical significance when compared to negative control VLRB MM3. Based on the robust signals obtained with VLRB B39 tetramers, we selected this VLRB tetramer for subsequent experiments exploring the detection of SARS-CoV-2 viral particles. Similar to the experiments using cell lysates from SARS-CoV-2 S-protein-expressing cells, SARS-CoV-2 viral particles were readily detected by VLRB B39 tetramers but not by VLRB MM3 tetramers at viral titres of 2x10^7^ and 4x10^6^ TCID_50_/ml (**Figure 6C**). These experiments demonstrate the rapid detection of SARS-CoV-2 in a single-step VLRB antibody-based detection system.

## Discussion

The non-conventional VLR antibody platform of jawless vertebrates provides a system of structurally distinct antigen receptors with highly specific antigen recognition characteristics constituting a repertoire whose magnitude rivals that of the immunoglobulin-based antibody systems of jawed vertebrates. In the present study we explored whether we could harness this platform to isolate monoclonal VLRB antibodies with SARS-CoV-2 S-protein specificity. Our characterization of the VLRB B7, VLRB B33, and VLRB B39 monoclonal VLRB antibodies confirmed that the highly specific VLRB reagents recognize the SARS-CoV-2 S-protein but not the S-proteins of related coronaviruses. Interestingly, the LRRv units - typically the regions of greatest sequence diversity - of the VLRB B7 and VLRB B39 antibodies are identical whereas the remaining LRR units and especially the C-terminal loop show numerous non-conserved residues. The similar antigen binding and virus neutralization characteristics of VLRB B7 and VLRB B39 raise the possibility that antigen recognition is primarily mediated by residues located within the three identical LRRv units. Structural analyses of VLRB B7 and VLRB B39 in complex with the S-protein antigen will shed light on this possibility. It is tempting to surmise that the 72 amino acids encoded by the LRRv units could form the basis for the development of a small peptide-based inhibitor interfering with the binding of SARS-CoV-2 to the ACE2 receptor. Structural analyses will also allow a comparison of potential differences among the epitopes recognized by VLRB B7 and VLRB B39 and neutralizing antibodies isolated from COVID-19 convalescent individuals, knowledge that will be informative in future vaccine design studies targeting SARS-CoV-2. Although the precise epitopes recognized by the VLRB antibodies remain to be determined, reactivity testing with the B.1.1.7 and B.1.351 variants provided important initial clues. The differential recognition of the B.1.1.7 and B.1.351 S-protein variants indicates that VLRB B7 and B39 recognize an epitope located within the RBD that involves residues K417 or E484 but not residue N501, which is mutated in both variants. The epitope recognized by VLRB B33 is more difficult to predict but is unlikely to involve residue D614, the only position located outside of sequences encoding the RBD that is mutated in both S-protein variants. The ability of VLRB B33 to detect S-protein in flow cytometry assays but not by ELISA further indicates that VLRB B33 may potentially recognize subtle differences between cell surface and soluble trimeric S protein. In control experiments using human serum collected 4 weeks after vaccination we observed noticeably weaker binding to both S-protein variants relative to the wild-type S-protein. While this could represent lower cells surface S-protein expression levels, the equal signal intensities observed for wt and B.1.1.7 variant S-protein using VLRB B39 for detection suggests that the vaccine-induced antibody response was less efficient in variant S-protein recognition.

The potent SARS-CoV-2 neutralizing activity of VLRB B7 and VLRB B39 raises the possibility of potential therapeutic applications of these VLRB antibodies. The observed IC50 values of 54.9ng/ml and 95.9ng/ml are comparable with those of numerous neutralizing human monoclonal antibodies isolated from COVID-19 convalescent individuals (4, 42, 43). In control experiments using the human monoclonal 4A8 antibody we observed IC50 values similar to those in the published literature (30), demonstrating comparability of our experimental system. Various human monoclonal antibodies are in development for therapeutic targeting of SARS-CoV-2 (8) and non-conventional SARS-CoV-2 reactive nanobodies isolated from immunized camelids are proposed for prophylactic or therapeutic reagents that can be applied as aerosols (44, 45). It will be important to investigate in animal models whether intranasal application of VLRB B7 or VLRB B39 reduces the rate of SARS-CoV-2 infection. While strategies to reduce potential immunogenicity of the VLRB antibodies may need to be considered, studies on ‘repebodies’, binding scaffolds designed on consensus LRR sequences of jawless vertebrate VLR proteins, showed remarkably low levels of immunogenicity (46).

Our demonstration that VLRB B39 tetramers function as single-step reagents that permit the rapid and sensitive detection of SARS-CoV-2 suggests that VLRB B39 is a promising candidate for development as a novel diagnostic tool. VLRB B39 did not cross-react with any of the tested S- proteins of other coronaviruses, including the most closely related SARS-CoV, indicating the high level of specificity of the interaction of this monoclonal VLRB antibody with the SARS-CoV-2 S-protein. The tetramer design of monomeric biotinylated VLRB B39 antigen binding units coupled to streptavidin-HRP allowed the reliable detection of SARS-CoV-2 of 2.8x10^6^ TCID_50_/ml, corresponding to calculated 1.4x10^5^ TCID_50_ in the 50µl reactions used in our experimental single step reactions. Further optimizations using different capture antibodies or enhanced sensitivity substrates for the HRP-coupled VLRB B39 tetramers are expected to increase assay sensitivity. RT-PCR analyses from samples of the upper respiratory tract (nasopharyngeal swabs and throat swabs) as well as from sputum and stool revealed SARS-CoV-2 concentrations ranging from 1x10^3^ – 1x10^11^ viral RNA copies/swab in samples taken between two days and three weeks post onset of symptoms (47, 48). These viral concentrations suggest that the detection limit of VLRB tetramers are within the range required for the detection of SARS-CoV-2 from clinical samples. Targeting epitopes on the SARS-CoV-2 S-protein has the additional advantage of avoiding the use of lysis reagents required for the use of reagents targeting the more abundant matrix components of the virus.

In conclusion, we describe the isolation of three monoclonal VLRB antibodies as novel reagents targeting SARS-CoV-2 with unique properties for biomedical research applications as well as clinical diagnostic and prophylactic potential. Beyond the current pandemic, it is possible that SARS-CoV-2 may become a circulating pathogen similar to the four endemic coronaviruses HCoV-OC43, HCoV-NL63, HCoV-HKU1, and HCoV-229E. VLRB antibodies exemplified by VLRB B7, B33, and B39 represent a new class of reagents that can complement conventional immunoglobulins to address this public health challenge.

## Data Availability Statement

The original contributions presented in the study are included in the article/**Supplementary Material**. Further inquiries can be directed to the corresponding author.

## Ethics Statement

The animal study was reviewed and approved by Animal Care Committee, University of Toronto.

## Author Contributions

LL, SK, PB, ZL, SG, YL, SD, JC, and GE conducted experiments. LL, PB, ZL, JR, MO, and GE conceived experimental designs and analyzed experimental data. LL and GE wrote the manuscript. All authors contributed to the article and approved the submitted version.

## Funding

This study was supported by CIHR grant MM1-17488 to GE. JC was supported by CIHR grants MOP106491 and PJT159450. JR was supported by CIHR grant OV3-170649.

## Conflict of Interest

The authors declare that the research was conducted in the absence of any commercial or financial relationships that could be construed as a potential conflict of interest.

## Abbreviations

HCoV, human coronavirus.
