# Supplementary material for: Detection and Neutralization of SARS-CoV-2 Using Non-conventional Variable Lymphocyte Receptor Antibodies of the Evolutionarily Distant Sea Lamprey
Source: Front Immunol. 2021 Jun 21;12:659071. doi: 10.3389/fimmu.2021.659071 (PMC8256154; doi:10.3389/fimmu.2021.659071)
Supplement: Supplementary file 1 [file DataSheet_1.pdf]

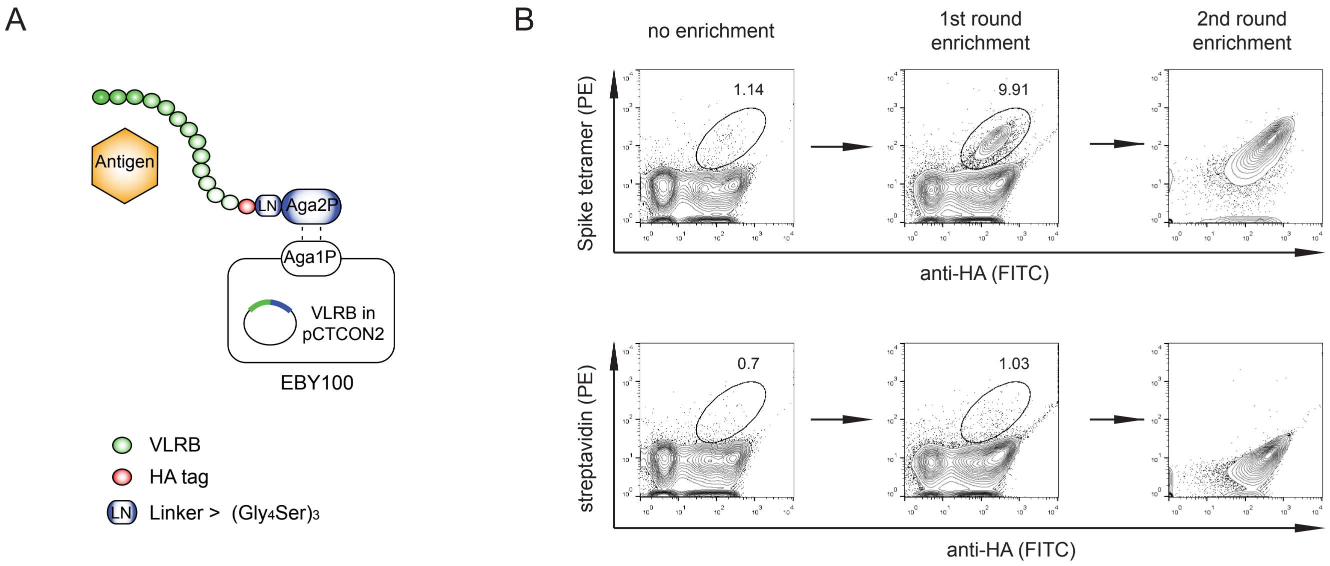

Supplementary Figure 1: Yeast surface display (YSD) library cloning and enrichment approach. (A) Schematic of YSD library design. Fusion proteins consisting of VLRB antigen binding domains lacking the C-terminal stalk regions are separated from the Aga2P fusion partner by the HA-epitope tag and a flexible linker. **(B)** Enrichment of SARS-CoV-2 S-protein reactive EBY100 cells following two enrichment cycles using S-protein/streptavidin-PE tetramers. Antigen reactivity was determined by incubation of yeast cultures following 24h culture in 1.9% galactose/0.1% dextrose with S-protein/streptavidin-PE tetramers (top row) or uncoupled streptavidin-PE (bottom row) in combination with anti-HA epitope antibodies to ascertain yeast cell surface expression of the VLRB library proteins.
