# Supplementary material for: Detection and Neutralization of SARS-CoV-2 Using Non-conventional Variable Lymphocyte Receptor Antibodies of the Evolutionarily Distant Sea Lamprey
Source: Front Immunol. 2021 Jun 21;12:659071. doi: 10.3389/fimmu.2021.659071 (PMC8256154; doi:10.3389/fimmu.2021.659071)
Supplement: Supplementary file 2 [file DataSheet_2.pdf]

A

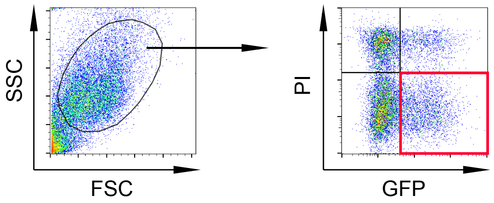

B

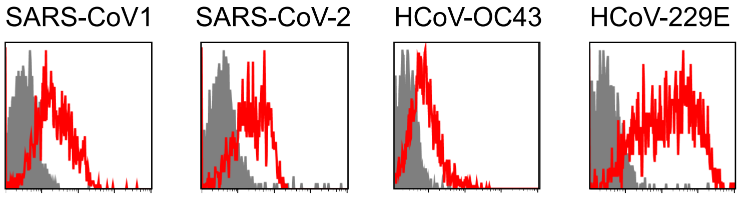

Supplementary Figure 2: Gating strategy and verification of cell surface expression of coronavirus Spike proteins. (A) HEK293F cells transiently co-transfected with coronavirus S-protein and GFP expression constructs were gated on the forward scatter (FSC) and side scatter (SSC) defined cell population, followed by propidium iodide and GFP signal gating. GFP-positive and PI-negative cell populations (red gate) were analyzed for VLRB antibody reactivity. **(B)** Validation of cell surface expression of SARS-CoV, SARS-CoV-2, HCoV-OC43 and HCoV-229E S-proteins in transiently transfected HEK293F cells. Cell gating was performed as displayed in (A). Open red histograms depict signals obtained with the indicated S-protein specific monoclonal antibody and filled grey histograms depict signals obtained with PE-labeled secondary antibodies only.
