# Supplementary material for: Detection and Neutralization of SARS-CoV-2 Using Non-conventional Variable Lymphocyte Receptor Antibodies of the Evolutionarily Distant Sea Lamprey
Source: Front Immunol. 2021 Jun 21;12:659071. doi: 10.3389/fimmu.2021.659071 (PMC8256154; doi:10.3389/fimmu.2021.659071)
Supplement: Supplementary file 3 [file DataSheet_3.pdf]

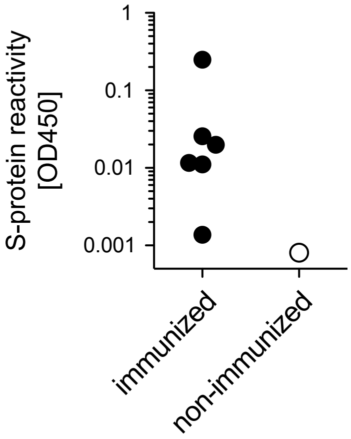

Supplementary Figure 3: Immune response of immunized sea lamprey larvae. Sea lamprey larvae were immunized with recombinant trimeric SARS-CoV-2 S-protein coated to Jurkat carrier cells. Reactivity of anti-sera from immunized animals (closed circles) and a non-immunized control animal (open circle) to recombinant S-protein was determined by ELISA. Anti-sera were diluted 1:10 in 0.66xPBS and incubated with antigen in S-protein coated wells of an ELISA plate, followed by incubation with mouse monoclonal anti-VLRB antibody clone 4C4 and detection with HRP-labeled anti-mouse Ig secondary reagents. 5 out of 6 immunized animals displayed noticeable S-protein reactivity.
