# Supplementary material for: Detection and Neutralization of SARS-CoV-2 Using Non-conventional Variable Lymphocyte Receptor Antibodies of the Evolutionarily Distant Sea Lamprey
Source: Front Immunol. 2021 Jun 21;12:659071. doi: 10.3389/fimmu.2021.659071 (PMC8256154; doi:10.3389/fimmu.2021.659071)
Supplement: Supplementary file 4 [file DataSheet_4.pdf]

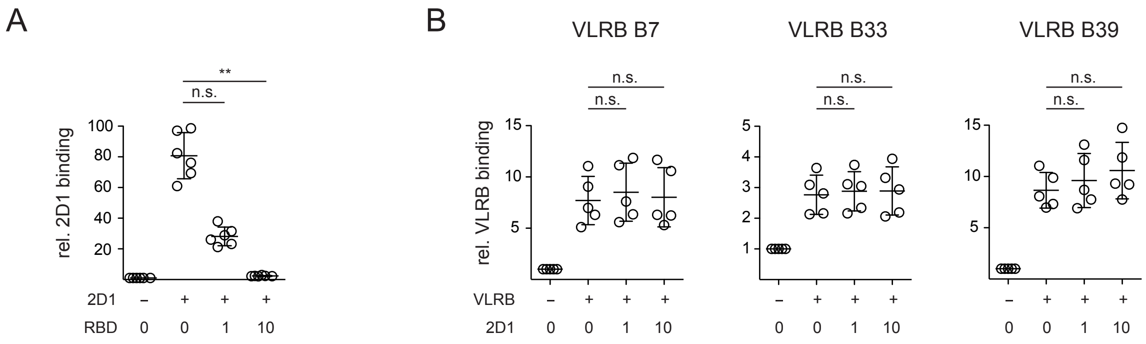

Supplementary Figure 4: Antibody 2D1 recognizes an epitope located within the SARS-CoV-2 RBD but does not compete with VLRB antibody binding. (A) Antibody 2D1 (1μg/ml) was pre-incubated with or without the indicated molar excess of recombinant RBD prior to addition to 293F cells expressing cell surface SARS-CoV-2 S-protein and assessment of 2D1 binding by flow cytometry. Symbols depict relative MFI values normalized to control experiments without 2D1 addition and horizontal bars indicate mean +/- SD. **(B)** VLRB B7, B33 or B39 antibodies (0.1μg/ml) were pre-incubated with or without the indicated molar excess of recombinant antibody 2D1 prior to addition to 293F cells expressing cell surface SARS-CoV-2 S-protein and assessment of VLRB antibody binding by flow cytometry. Symbols depict relative MFI values normalized to control experiments without VLRB addition and horizontal bars indicate mean +/- SD. Statistical significance was determined using Friedman tests (A, n=6), (B, n=5) with Dunns post hoc tests and is indicated by asteriscs (**) for $p<0.01$ and (n.s.) for non-significant.
